# OpenAI single-agent LLM architecture reduces computational overhead relative to multi-agent orchestration in a simulated mars rover decision-support benchmark

**DOI:** 10.3389/frobt.2026.1877762

**Published:** 2026-07-06

**Authors:** Dan Sanabria

**Affiliations:** Type 3 Innovations, LLC, Alexandria, VA, United States

**Keywords:** AI benchmarking, decision support systems, hazard detection, large language models, LLM agents, mars rover autonomy, multi-agent orchestration, planetary robotics

## Abstract

Mars rover missions require decision-support systems that can interpret terrain, telemetry, environmental conditions, and mission objectives under delayed communication with Earth. This study evaluates whether multi-agent orchestration improves simulated Mars rover decision support compared with a single-agent baseline. A controlled benchmark of 100 synthetic mission-inspired rover scenarios was evaluated using OpenAI GPT-4o and GPT-5.5, with five repeated runs per scenario and architecture. Model-facing scenario inputs were separated from evaluator-side labels so that expected actions and hazards were reserved for scoring only. Performance was measured using decision accuracy, exact and substring-based semantic hazard F1, hazard error counts, latency, token usage, scenario-level paired statistical comparisons, and GPT-4o specialist-agent ablations. Across the tested OpenAI configurations, the single-agent architecture showed numerical advantages in decision accuracy and hazard-label alignment, but these decision-quality differences were not consistently significant under scenario-level statistical analysis with Holm-Bonferroni adjustment. The only decision-quality metric remaining significant was GPT-5.5 exact hazard F1, although absolute values were very low. The most reliable difference was computational efficiency: the single-agent architecture required substantially lower latency and token usage than the prompt-defined multi-agent orchestration architecture. Multi-agent orchestration generated broader hazard lists, including plausible non-canonical observations, but did not reliably improve aggregate decision accuracy or hazard F1. These findings suggest that, for short-context, tool-less, static decision-support tasks where all relevant context is available in a single input, multi-agent orchestration should be treated as a cost-bearing design choice rather than an assumed improvement. The study contributes a reproducible architecture-level benchmark for evaluating when LLM-based orchestration is worth its operational cost in mission-inspired workflows.

## 1 Introduction

Planetary surface exploration depends on robotic systems that can interpret uncertain terrain, manage constrained resources, and support safe navigation decisions under delayed communication with Earth. Mars rover missions are especially affected by this challenge because mobility decisions must account for terrain composition, slope, wheel-soil interaction, battery state, thermal limits, communication delay, mission objectives, and science-priority tradeoffs. Although modern rover autonomy has improved surface traversal, human operators still play a central role in planning, reviewing, and approving mission-critical activities.

Large language models (LLMs) offer a potential mechanism for structured decision support because they can reason over heterogeneous mission context, synthesize natural-language observations, and generate explainable recommendations. In this setting, an LLM-based decision-support system can be prompted with structured rover scenario data and asked to recommend an operational action, identify hazards, and explain its reasoning. However, general-purpose LLM capability does not automatically imply reliable performance in constrained mission-inspired settings. Outputs must remain consistent, grounded in the provided scenario, aligned with expected operational actions, and efficient enough to support repeated analysis of mission records.

A central architectural question is whether multi-agent orchestration improves decision-support quality enough to justify its added complexity. Multi-agent systems are often motivated by the assumption that task decomposition enables specialist reasoning. For Mars rover decision support, separate agents could evaluate telemetry, terrain, risk, and mission-planning factors before an orchestrator synthesizes their outputs. This structure may improve interpretability, but it can also introduce coordination overhead, redundant risk language, inconsistent intermediate outputs, higher latency, higher token usage, and broader hazard enumeration that does not necessarily improve the final decision.

This study evaluates that architectural tradeoff through a controlled synthetic Mars rover decision-support benchmark. The benchmark compares a single-agent baseline against a prompt-defined multi-agent orchestration architecture across 100 mission-inspired scenarios with five repeated runs per condition. Each scenario requires the model to recommend one of four operational actions: proceed, pause, reroute, or request human review. The benchmark also evaluates hazard identification using exact canonical-label matching and deterministic substring-based semantic matching, along with latency, token usage, false-positive hazards, false-negative hazards, and scenario-level behavior.

The study focuses on two OpenAI model configurations, GPT-4o and GPT-5.5. These models were selected to evaluate whether architectural effects persist across different model backends from the same provider. The study does not claim to evaluate all LLM providers, all model families, or all possible multi-agent designs. Instead, it examines whether a supervisor-style multi-agent orchestration workflow provides measurable benefits over a single-agent baseline in a compact, fully contextualized synthetic decision-support task.

The benchmark was designed to separate model-facing scenario inputs from evaluator-side scoring labels. Expected actions and expected hazards were reserved for evaluation and excluded from prompts and model-facing payloads. This separation is important because the study evaluates whether the model can infer an appropriate rover action and hazard set from operational scenario context, not whether it can reproduce labels exposed in the input. Prompt templates, model-facing fields, output schema, and run metadata are provided in the supplementary materials to support reproducibility.

The study evaluates whether multi-agent orchestration improves hazard identification, increases latency and token usage, changes action-selection consistency across model backends, or shifts error patterns such as risk tolerance, conservative escalation, or broader hazard enumeration. These expectations are treated as empirical questions rather than presumed outcomes. By evaluating decision accuracy, hazard F1, false-positive and false-negative hazards, latency, token usage, paired statistical comparisons, and ablation results, this work contributes a reproducible benchmark for assessing when LLM-based orchestration is worth its operational cost in mission-inspired decision-support settings.

## 2 Related work

### 2.1 Autonomous robotics and planetary exploration

The analytical framing for this study draws from a focused set of foundational works in Mars rover autonomy, sensing, machine learning for space robotics, distributed autonomy, and constrained computation. Rather than incorporating the full dissertation reference base, this paper uses a smaller subset of prior work to establish the operational and technical conditions motivating the present benchmark.

Autonomous planning and safety-critical decision-making in planetary systems are informed by Abcouwer et al. (2021), Brockers et al. (2021), Sasaki et al. (2020), and Verma et al. (2023). These works establish the relevance of perception-driven planning, rover navigation, aerial/rover coordination, and autonomy under mission-relevant constraints. They support the central premise that autonomous systems for Mars exploration must be evaluated not only by task success, but also by reliability, environmental uncertainty, and operational feasibility.

Sensing and perception constraints are grounded in Beegle et al. (2015), Maki et al. (2020), and Wilhelm et al. (2022). These studies provide context for rover instrumentation, *in situ* sensing, imaging systems, and machine-learning challenges associated with Mars-like remote sensing environments. Their relevance to the present study lies in the fact that LLM-based decision-support systems must reason from structured scenario inputs and mission logs that approximate delayed, incomplete, and operationally constrained observations.

Broader AI and autonomy concepts are supported by Arzo et al. (2023) and Ferreira et al. (2017), which address space exploration technologies, machine learning, and AI-enabled communication or decision-support concepts under aerospace constraints. These works help position the present benchmark within a wider research trajectory focused on AI-enabled space operations.

Distributed and multi-agent autonomy concepts are informed by Dimakos et al. (2021) and Omar et al. (2024), which examine decentralized swarm robotics and space-oriented swarm systems. Although this paper does not evaluate robotic swarms directly, these works provide relevant background for comparing centralized single-agent reasoning against distributed multi-agent decomposition.

Finally, constrained and emerging computation is represented by Barnell et al. (2023) and Murbach and Salas (2022), which highlight the importance of energy-efficient and edge-compatible computing approaches for autonomous systems. These works reinforce the need to evaluate LLM agent architectures not only by decision quality, but also by latency, token consumption, and computational overhead.

Together, these selected foundational works establish the Mars autonomy, sensing, AI, distributed-system, and constrained-computation context for this study. The present paper narrows that foundation into a controlled empirical comparison of single-agent and multi-agent LLM architectures for simulated Mars rover decision support.

### 2.2 Large language models for decision support

The LLM component of this study is grounded in foundational work on transformer-based language modeling and foundation models. Vaswani et al. (2017) introduced the Transformer architecture, establishing the attention-based modeling foundation underlying contemporary large language models. Brown et al. (2020) demonstrated that large autoregressive language models can perform a range of tasks through few-shot prompting without task-specific fine-tuning. Bommasani et al. (2021) further framed such systems as foundation models, emphasizing their broad adaptability, emergent capabilities, and downstream risks.

These works support the present study’s use of general-purpose LLMs as decision-support systems rather than task-specific models trained only for rover autonomy. They also motivate the need to evaluate model behavior empirically because broad adaptability does not guarantee reliable performance in constrained mission-inspired decision environments.

Recent work on LLM-based multi-agent systems is directly relevant to the architecture comparison in this paper. Liu et al. (2025) examine conformity in reasoning LLMs within multi-agent systems and show that reasoning-oriented models may reduce but do not eliminate conformity, particularly across contextual tasks. This is relevant to the present benchmark because multi-agent orchestration may introduce consensus-seeking or conservative behavior that does not necessarily improve decision quality.

Das et al. (2025) provide a current implementation-oriented discussion of multi-agent system architectures, including network-based, supervisor-led, and hierarchical designs. Their discussion of specialization, modularity, control flow, and inter-agent communication provides useful context for the specialist-agent plus orchestrator design evaluated in this study. Similarly Du et al. (2026) examine decentralized multi-agent reinforcement learning under limited communication, partial observability, and goal-oriented coordination constraints. Although their work is not LLM-specific, it supports the broader argument that multi-agent coordination must be evaluated with attention to communication relevance, scalability, and operational constraints.

### 2.3 LLM-agent benchmarks and orchestration frameworks

Recent work on LLM-agent benchmarking provides additional context for the present study. AgentBench evaluates large language models as agents across interactive environments, emphasizing the need to test agent behavior through task execution rather than static language capability alone (Liu et al., 2023). ToolLLM and ToolBench extend this direction by evaluating LLMs in tool-use and API-oriented settings, showing that agent performance depends not only on model capability but also on instruction following, tool selection, and execution reliability (Qin et al., 2023). MLAgentBench similarly evaluates language agents in iterative machine-learning experimentation, highlighting the importance of repeated task execution, workflow structure, and measurable performance outcomes (Huang et al., 2023).

Multi-agent orchestration frameworks also inform the present architecture comparison. AutoGen demonstrates how LLM applications can be structured through multi-agent conversation patterns, role assignment, and coordinated workflows (Wu et al., 2023). ReAct provides a related foundation for combining reasoning and acting in language models, supporting the broader idea that LLM systems can generate intermediate reasoning steps before producing task-directed actions (Yao et al., 2022). Together, these works show that LLM-agent systems are increasingly evaluated not only as text generators, but as structured decision-making or task-execution systems.

Structured-output reliability is also relevant to LLM-agent benchmarks because decision-support systems often require machine-readable outputs that can be parsed and scored consistently. Recent work on structured generation and constrained decoding shows that JSON-schema-constrained outputs can improve parseability and evaluation consistency, but also require explicit assessment of output validity, efficiency, schema coverage, and downstream answer quality (Geng et al., 2025).

The present benchmark differs from these prior agent benchmarks in scope and domain. It does not evaluate general tool use, software engineering, or open-ended interactive environments. Instead, it evaluates whether a prompt-defined single-agent or multi-agent orchestration architecture produces better rover decision-support outputs under a controlled synthetic Mars scenario benchmark. This distinction is important because mission-inspired decision support requires evaluating action selection, hazard identification, latency, token usage, and failure behavior under repeated and matched scenario conditions. The present study therefore complements existing LLM-agent benchmarks by focusing on architecture-level tradeoffs in a constrained, safety-relevant decision-support setting.

### 2.4 Multi-agent orchestration systems

Multi-agent orchestration systems decompose complex tasks across specialized agents and use a coordinating mechanism to synthesize intermediate outputs into a final response. In LLM-based systems, these architectures often appear as supervisor-led, hierarchical, or network-based workflows in which agents exchange intermediate analyses before a final recommendation is produced. Their main appeal is that specialization may improve reasoning quality, modularity, and interpretability.

These potential benefits are relevant to Mars rover decision support because telemetry interpretation, terrain assessment, mission planning, and risk evaluation represent distinct analytical functions. A multi-agent design can assign each function to a specialist agent and then use an orchestrator to combine the outputs into a final operational recommendation. In principle, this structure allows each specialist contribution to be inspected before final synthesis.

However, decomposition also introduces tradeoffs. Each additional agent call can increase latency, token consumption, and implementation complexity. Intermediate outputs may also contain overlapping, inconsistent, or overly cautious interpretations that must be reconciled by the orchestrator. In mission-inspired decision environments, this can produce unnecessary pauses, reroutes, or human-review requests if risk-related language is amplified without improving correctness.

For this reason, multi-agent orchestration should not be assumed to improve decision quality simply because it increases architectural complexity. The relevant empirical question is whether task decomposition improves reliability enough to justify its operational costs. In this study, specialist agents evaluate telemetry, terrain, risk, and mission-planning factors before an orchestrator produces the final recommendation, allowing direct comparison with a single-agent baseline that receives the same complete scenario context.

### 2.5 Research gap

Existing literature provides substantial foundations in planetary autonomy, rover navigation, sensing, machine learning for space systems, foundation models, LLM-agent benchmarking, tool-use evaluation, and multi-agent coordination. However, fewer studies directly evaluate whether multi-agent LLM orchestration improves decision-support reliability enough to justify the additional latency, token usage, and coordination complexity introduced by the architecture. Much of the multi-agent and LLM-agent benchmark literature focuses on general interactive environments, tool use, software or machine-learning workflows, robotic swarms, reinforcement learning, distributed control, or general collaborative LLM behavior rather than controlled comparisons of single-agent and multi-agent LLM systems under mission-inspired rover decision-support conditions.

This gap is important because mission-support environments require more than high-level reasoning quality. A useful decision-support system must also maintain consistency, avoid unnecessary hazard inflation, limit false positives and false negatives, and produce outputs within practical latency and computational-cost boundaries. In a constrained operational context, an architecture that produces marginally better reasoning, but substantially greater latency or escalation behavior may not represent an effective tradeoff.

The present study addresses this gap by evaluating single-agent and multi-agent LLM architectures on a labeled benchmark of 100 synthetic Mars rover scenarios with repeated runs. Each architecture is evaluated on the same decision task: selecting whether the rover should proceed, pause, reroute, or request human review based on telemetry, terrain context, mission objectives, and mission-log information. The benchmark measures decision accuracy, exact and semantic hazard F1, hazard false positives and false negatives, latency, token usage, confidence, scenario-level behavior, and paired statistical comparisons.

By combining mission-inspired synthetic scenarios, structured JSON outputs, repeated evaluations, and architecture-level statistical comparisons, this study provides a controlled empirical basis for assessing whether multi-agent decomposition improves rover decision support or primarily introduces coordination overhead. The contribution is therefore not a new rover autonomy controller, but an architecture-level benchmark for evaluating LLM decision-support behavior under conditions inspired by planetary robotic operations.

## 3 Materials and methods

### 3.1 Study intent and experimental design

This benchmark evaluates whether large language model (LLM) agent architecture influences decision-support performance in simulated Mars rover operations. The codebase is designed to test a focused scientific question: when provided with the same mission scenario information, does a single generalized agent produce more reliable and efficient rover recommendations than a multi-agent orchestration system that decomposes the task across specialized agents? This comparison is meaningful because multi-agent systems are often proposed as a way to improve reasoning through specialization, yet such decomposition may also introduce coordination overhead, redundant risk interpretation, inconsistent hazard synthesis, and increased latency.

Synthetic but labeled Mars rover scenarios are used to create a controlled benchmark in which model outputs can be evaluated against known expected actions and canonical hazard labels. These labels do not represent operational ground truth from a deployed rover; rather, they provide a repeatable evaluation basis for comparing architectural behavior under mission-inspired decision conditions.

The objective of the experimental method is to provide a repeatable benchmark for comparing single-agent and multi-agent LLM decision-support architectures under simulated Mars rover operating conditions. For each scenario, the system must recommend one operational action: proceed, pause, reroute, or request human review. The benchmark evaluates both the correctness of the recommended action and the quality of hazard identification using exact and semantic matching metrics. In addition to decision quality, the experiment measures latency and token usage to determine whether orchestration provides sufficient reliability benefit to justify its added computational cost. Repeated runs are used to assess consistency across identical scenarios, while paired statistical comparisons are used to evaluate differences between architectural conditions.

### 3.2 Scenario construction and labeling

The benchmark used 100 synthetic Mars rover decision-support scenarios. The scenario corpus was generated using a deterministic generation script and template-based scenario construction. The scenarios were designed to represent mission-inspired rover decision contexts involving terrain conditions, vehicle-state indicators, environmental factors, mission objectives, expected rover actions, and expected hazards. The scenario count was limited to 100 due to API cost, runtime, and repeatability constraints. This size was selected to provide a compact but structured benchmark that could be repeated across multiple model and architecture conditions while remaining feasible for iterative experimentation.

To reduce direct author influence on individual scenario outcomes, the scenario set was not exhaustively hand-curated scenario-by-scenario prior to benchmarking. This was intended to limit *post hoc* adjustment of the scenario set based on expected model behavior. However, this also means that the scenario labels should be interpreted as benchmark labels rather than independently validated operational ground truth. The scenarios were synthetic and mission-inspired; they were not derived from live rover telemetry or validated flight operations. As a result, the benchmark evaluates model behavior against a predefined synthetic decision policy rather than against mission-certified rover-control labels.

The scenario templates were designed to vary terrain conditions, rover-state variables, environmental factors, mission objectives, action classes, and hazard categories so that the benchmark would include multiple decision contexts rather than repeated versions of a single case type. This variation was intended to reduce dependence on a narrow scenario pattern and to support repeated architecture-level comparisons across different operational conditions. However, the scenario distribution was controlled synthetically rather than derived from operational rover telemetry or independently validated mission data. Therefore, the benchmark supports architecture-level comparison under repeatable simulated conditions, but it should not be interpreted as proving robustness across the full distribution of real Mars terrain, telemetry, and mission-planning conditions.

The benchmark was subsequently executed through the OpenAI API using the selected OpenAI model backends. The models evaluated in the benchmark were separate from any systems used to assist with code development and scenario generation. The benchmark evaluations themselves were generated through the OpenAI API runs recorded in the run metadata.

### 3.3 Experimental conditions and dataset

The study compares two primary architectural conditions. In the single-agent architecture, one generalized LLM agent receives the complete rover scenario context and independently generates a final operational recommendation. In the multi-agent orchestration architecture, specialized agents independently evaluate telemetry, terrain, risk, and mission-planning factors before forwarding intermediate outputs to an orchestration layer responsible for synthesizing the final recommendation.

The experimental dataset consists of 100 simulated Mars rover mission scenarios. Each scenario includes structured mission-relevant inputs such as rover telemetry, terrain context, mission objectives, environmental conditions, operational hazards, and mission logs. Each scenario is evaluated across five repeated runs per architecture condition, producing 500 evaluations per architecture for each model configuration.

The experiment evaluates OpenAI GPT-4o and GPT-5.5 model configurations. OpenAI models were selected as the LLM provider for this benchmark to support consistent access to general-purpose model configurations. The study does not compare all available commercial or open-weight LLM providers, and the findings should not be interpreted as a comprehensive vendor comparison.

### 3.4 Prompt inputs and leakage controls

To address the risk of label leakage, model-facing scenario inputs were separated from scoring-only evaluation labels. The full scenario record included expected actions and canonical expected hazards for evaluation purposes, but these fields were not intended to be available to any model during response generation. In the revised benchmark workflow, expected_action and expected_hazards were treated as scoring-only fields and removed from model-facing payloads before calls to the single-agent model, specialist agents, and orchestrator agent.

This separation ensured that each architecture generated recommendations from operational scenario context rather than from ground-truth labels. The single-agent architecture received the sanitized operational scenario context directly. In the multi-agent orchestration condition, each specialist agent received only the operational fields relevant to its role, and the orchestrator received sanitized scenario context along with the specialist outputs. The original full scenario record was retained only for evaluator-side scoring after the model response was generated.

The benchmark code also included validation checks to ensure that scoring-only fields were not present in model-facing payloads. These checks were added to prevent expected_action and expected_hazards from being accidentally included in prompts or structured payloads. This design supports a like-for-like comparison between the single-agent and multi-agent orchestration conditions by ensuring that both architectures operate without privileged access to the expected outputs used for scoring.

### 3.5 Prompt documentation and reproducibility

To support reproducibility and address prompt-dependence in the benchmark, the full prompt templates used for the single-agent baseline, telemetry agent, terrain agent, risk agent, mission-planner agent, and orchestrator agent are provided in the Supplementary Material. The Supplementary Material also lists the model-facing scenario fields passed to each agent and distinguishes those operational fields from scoring-only fields used exclusively by the evaluator.

This documentation is important because the comparison evaluates prompt-defined agent architectures rather than independently trained expert models. Differences between the single-agent and multi-agent orchestration conditions may therefore reflect both architectural structure and prompt design. Providing the prompts, model-facing fields, output schema, and run metadata enables replication and allows readers to evaluate whether observed performance differences are attributable to orchestration behavior, role definitions, or implementation choices.

### 3.6 Experimental workflow

**FIGURE 1:**
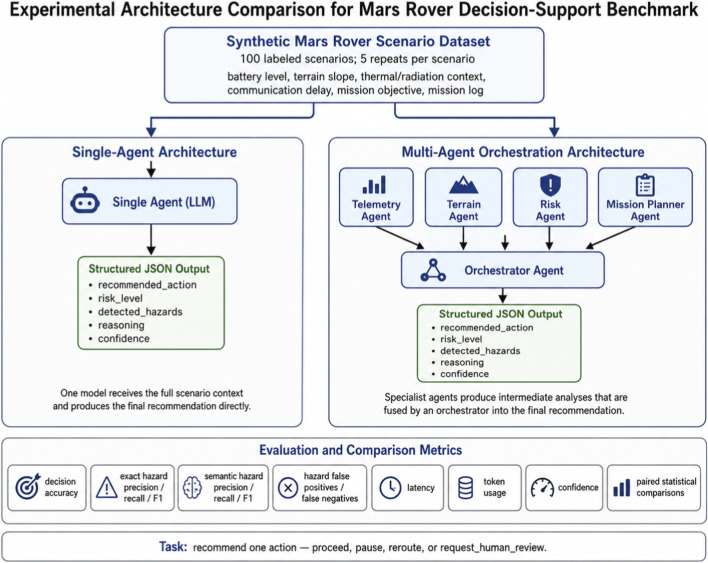
Experimental architecture comparison for the Mars rover decision-support benchmark. The benchmark compares a single-agent architecture, in which one LLM receives the full synthetic rover scenario and produces a final structured JSON recommendation directly, with a multi-agent orchestration architecture, in which specialist agents for telemetry, terrain, risk, and mission planning generate intermediate analyses that are fused by an orchestrator agent into the final recommendation. Both architectures are evaluated on the same dataset of 100 labeled Mars rover scenarios with five repeated runs per scenario. Outputs are compared using decision accuracy, hazard precision, recall, F1, false positives and false negatives, latency, token usage, confidence, and paired statistical comparisons.

Figure 1 illustrates the benchmark architecture used in this study, including the shared synthetic Mars rover scenario dataset, the single-agent and multi-agent orchestration pipelines, and the evaluation metrics used for comparison. Both architectures receive the same structured scenario inputs, including rover telemetry, terrain context, thermal and radiation conditions, communication delay, mission objective, and mission-log information. The primary difference between conditions is therefore architectural rather than data-based: the single-agent condition routes the full scenario directly to one LLM, whereas the multi-agent orchestration condition decomposes the scenario across specialist agents before final synthesis by an orchestrator.

In each experimental run, the benchmark loads a scenario from the synthetic dataset and submits it to both architecture conditions. Each architecture returns a constrained structured JSON object containing the recommended action, risk level, detected hazards, reasoning, and confidence score. These outputs are then evaluated against the expected action and canonical expected hazard list assigned to the scenario.

The evaluation pipeline records both task-performance and systems-performance metrics. Task-performance metrics include decision correctness, exact hazard precision/recall/F1, semantic hazard precision/recall/F1, hazard false positives, and hazard false negatives. Systems-performance metrics include latency and token usage. The resulting outputs are aggregated into architecture-level and scenario-level summaries, followed by paired statistical comparisons between the single-agent and multi-agent orchestration conditions.

### 3.7 Outputs and evaluation metrics

Each architecture returns a constrained structured JSON object containing the recommended action, risk level, detected hazards, reasoning, and confidence score. These outputs are evaluated against the expected action and canonical expected hazard list assigned to each scenario.

The primary outcome metrics include decision correctness, exact hazard precision/recall/F1, semantic hazard precision/recall/F1, hazard false positives, hazard false negatives, and confidence score. Efficiency metrics include latency in seconds and token usage. Robustness and repeatability are assessed through repeated scenario evaluations, architecture-level summaries, scenario-level summaries, and paired statistical comparisons across architectures.

Exact hazard F1 evaluates whether detected hazards match the predefined canonical hazard labels exactly. Semantic hazard F1 evaluates whether detected hazards correspond to the same underlying hazard concepts when wording differs from the canonical label. Together, these metrics distinguish strict label-matching performance from concept-level hazard recognition. Precision reflects the proportion of detected hazards that were correct, while recall reflects the proportion of expected hazards identified by the model; F1 provides the harmonic mean of precision and recall.

### 3.8 Semantic hazard evaluation

Hazard detection was evaluated using both exact-match and semantic matching approaches. Exact matching measured whether the model reproduced the canonical expected hazard labels in the benchmark. This metric was intentionally strict and should be interpreted as label-fidelity scoring rather than a complete measure of operational hazard awareness.

Semantic hazard matching was used to provide a more flexible evaluation of whether a detected hazard described the same underlying risk concept as an expected hazard, even when the wording differed. This component used substring-based keyword matching to identify conceptual overlap. For example, a detected hazard such as “low battery level” was treated as semantically equivalent to the canonical label “low battery” if they shared substring content. Detected hazards that were not semantically matched to any expected hazard were further classified as either plausible non-canonical observations (if they contained keywords present in the scenario context such as terrain description, mission log, or science objective) or spurious hazards (if they lacked contextual support).

The statistical calculations and aggregate metric summaries were performed through Python analysis scripts. In this workflow, exact-match and substring-based semantic matching were performed deterministically via Python code, which also computed precision, recall, F1, decision accuracy, latency summaries, token summaries, paired comparisons, p-values, confidence intervals, and related aggregate outputs. This approach ensured reproducibility while distinguishing between strict label fidelity and conceptual hazard awareness.

### 3.9 Statistical analysis

Statistical comparisons were revised to address the repeated-run structure of the benchmark. Each model-architecture condition included 100 scenarios with five repeated evaluations per scenario. Because repeated evaluations from the same scenario are correlated, scenarios rather than individual runs were used as the primary unit of statistical inference. For each model and architecture condition, the five repeated observations for each scenario were aggregated into scenario-level means, yielding 100 paired scenario-level units for each single-agent versus multi-agent comparison.

Scenario-level paired statistical comparisons were then used to evaluate whether observed differences between the single-agent and multi-agent orchestration architectures were consistent across matched scenarios. For each paired comparison, the null hypothesis was that there was no architecture-level difference between the single-agent and multi-agent orchestration conditions for the metric being tested. The alternative hypothesis was that the paired metric difference between architectures was nonzero.

Exact paired sign tests were conducted on the scenario-level paired observations. Cohen’s dz was reported as a standardized effect size, with 95% bootstrap confidence intervals generated by resampling paired scenarios. This scenario-level resampling approach preserves the paired scenario structure and avoids treating the five repeated runs per scenario as independent units of inference. Holm-Bonferroni correction was applied across 10 comparisons, representing five metrics across two model backends.

Effect direction was interpreted according to the metric: higher values were preferred for decision correctness and hazard F1, while lower values were preferred for latency, token usage, and hazard false-positive counts. Statistical outputs were reported alongside practical effect sizes and confidence intervals to distinguish statistically detectable differences from differences that may or may not be operationally meaningful.

### 3.10 Ablation analysis

To further examine the contribution of specialist roles within the multi-agent orchestration architecture, an ablation analysis was conducted for the GPT-4o condition. In each ablation run, one specialist agent was removed from the multi-agent workflow while the remaining specialists and orchestrator continued to generate a final decision recommendation. The evaluated ablation conditions removed the telemetry agent, terrain agent, risk agent, or mission-planner agent one at a time.

This analysis was designed to determine whether the multi-agent architecture depended equally on all specialist roles or whether some agents contributed more strongly to decision quality. Ablation results were compared against the full GPT-4o multi-agent orchestration baseline using paired run-level McNemar comparisons matched by scenario_id and run_id for binary decision correctness. Because the ablation analysis was conducted only for GPT-4o and used run-level paired comparisons rather than the scenario-level aggregation used in the primary architecture comparisons, the ablation findings are interpreted as exploratory sensitivity analyses.

### 3.11 Scope and cost constraints

The study was intentionally limited in scope due to API cost and runtime constraints. The main benchmark evaluated two OpenAI model backends (GPT-4o and GPT-5.5) across 100 scenarios with repeated runs, while the ablation analysis was conducted exclusively for the GPT-4o condition. GPT-5.5 ablation runs were not performed because of substantially higher API cost and runtime overhead per condition. Therefore, the ablation findings should be interpreted as evidence about specialist-role contributions within the GPT-4o multi-agent configuration, not as a comprehensive ablation analysis across all tested model backends.

Future work should expand the scenario corpus, add independent human review of scenario labels, compare deterministic hazard scoring against human annotation, and run ablation studies across additional model backends. Additional studies should also evaluate whether the observed role-contribution patterns generalize to more expensive reasoning models, heterogeneous multi-agent systems, and larger scenario sets.

### 3.12 Scope and limitations

The dataset used in this study represents a controlled simulation environment rather than real Mars rover operations. The evaluated systems were not deployed on radiation-hardened hardware, onboard rover systems, or operational mission infrastructure. Current LLM systems require computational resources substantially beyond what is currently feasible for direct deployment aboard planetary robotic systems. Consequently, the study should be interpreted as an evaluation of experimental reasoning architectures operating on transmitted rover datasets rather than a validation of real-time autonomous Mars operations.

The expected actions and canonical hazard labels are designed evaluation targets, which means results may be sensitive to how those labels are defined. Model behavior may also vary based on prompt wording, output schema constraints, sampling settings, model version, and orchestration design. Because the multi-agent condition depends on intermediate specialist outputs and final synthesis, its performance may be particularly sensitive to agent role definitions, escalation rules, and arbitration logic.

This study uses general-purpose frontier LLMs rather than models distilled, fine-tuned, or post-trained specifically for Mars rover operations. This choice supports a controlled evaluation of architecture-level behavior using accessible model configurations, but it does not test whether domain-specialized training would improve rover decision quality, reduce hazard over-identification, or improve action consistency. Future work may extend the benchmark by evaluating distilled models, fine-tuned models, or domain-adapted post-training approaches; however, such work would require additional dataset preparation, training infrastructure, validation procedures, and safety evaluation.

These limitations do not invalidate the benchmark, but they frame it as a controlled simulation study intended to compare architectural behavior under consistent experimental conditions using general-purpose LLM systems.

## 4 Results

Unless otherwise noted, accuracy, precision, recall, and F1 values are reported to three decimal places; latency values are reported to two decimal places; token counts are rounded to the nearest whole token; and percentages are reported to one decimal place. Statistical comparisons are reported using rounded p-values, with very small values reported as *p* < 0.001.

### 4.1 Aggregate architecture performance

The benchmark separated operational scenario inputs from scoring-only evaluation labels. The expected_action and expected_hazards fields were excluded from all single-agent, specialist-agent, and orchestrator prompts and were retained only for evaluator-side scoring after model responses were generated. The results reported in this section are therefore based on the finalized no-label-leakage benchmark workflow.

Across the finalized runs, single-agent decision accuracy differed by model backend. GPT-4o reached a decision accuracy of 0.810 across 500 single-agent evaluations, while GPT-5.5 reached 0.974 across 500 single-agent evaluations. Hazard-label alignment was lower than decision accuracy for both models. GPT-4o produced an exact hazard F1 of 0.081 and a semantic hazard F1 of 0.131, while GPT-5.5 produced an exact hazard F1 of 0.018 and a semantic hazard F1 of 0.075. These results indicate that action selection remained stronger than hazard-label fidelity under both strict exact-match scoring and substring-based semantic hazard scoring.

Efficiency results showed that GPT-4o had lower mean latency and token usage than GPT-5.5 in the single-agent runs. GPT-4o averaged 2.32 s and 458 tokens per evaluation, while GPT-5.5 averaged 6.06 s and 548 tokens per evaluation. These values provide a baseline for interpreting backend-level cost-performance tradeoffs before architecture-level and ablation comparisons.

### 4.2 Aggregate architecture comparison

The experimental evaluation compared single-agent and multi-agent orchestration architectures across two OpenAI model configurations: GPT-4o and GPT-5.5. Each model run used 100 simulated Mars rover scenarios with five repeated evaluations per scenario, producing 500 evaluations per architecture condition for each model configuration. The reported results are based on the finalized no-label-leakage benchmark workflow, in which expected_action and expected_hazards were excluded from all model-facing payloads and retained only for evaluator-side scoring.

Decision accuracy was calculated as the proportion of evaluations in which the model’s recommended action exactly matched the scenario’s expected_action. Hazard metrics were calculated from detected_hazards compared with expected_hazards. Exact hazard F1 used strict normalized label matching, while semantic hazard F1 used deterministic substring-based matching to capture cases where the detected and expected hazards described the same underlying concept using different wording. Precision, recall, and F1 were then calculated from true positives, false positives, and false negatives. Latency and token usage were averaged across the 500 evaluations in each model and architecture condition.

**FIGURE 2:**
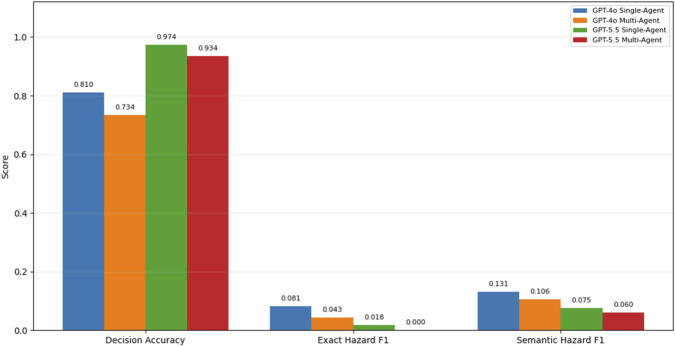
Decision quality metrics by model and architecture. Decision accuracy, exact hazard F1, and semantic hazard F1 are shown for the single-agent and multi-agent orchestration architectures across GPT-4o and GPT-5.5. Metrics are calculated across 500 evaluations per architecture condition. Decision accuracy was higher for the single-agent architecture in both model configurations, while hazard F1 scores remained low across architectures under strict canonical-label and substring-based semantic scoring.

**FIGURE 3:**
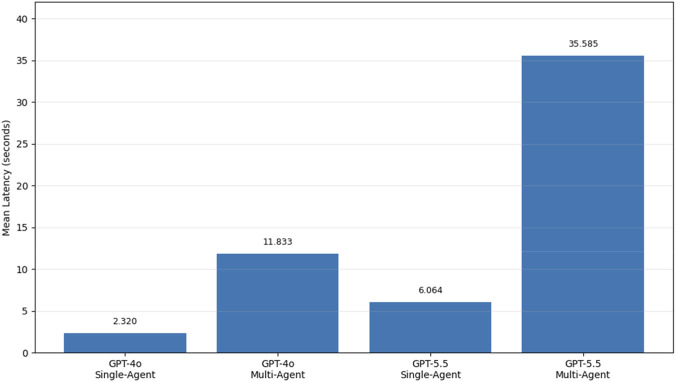
Mean latency by model and architecture. Mean response latency is shown for the single-agent and multi-agent orchestration architectures across GPT-4o and GPT-5.5. Multi-agent orchestration required substantially more processing time than the single-agent baseline in both model conditions, reflecting the added cost of multiple specialist-agent calls and final orchestration.

**FIGURE 4:**
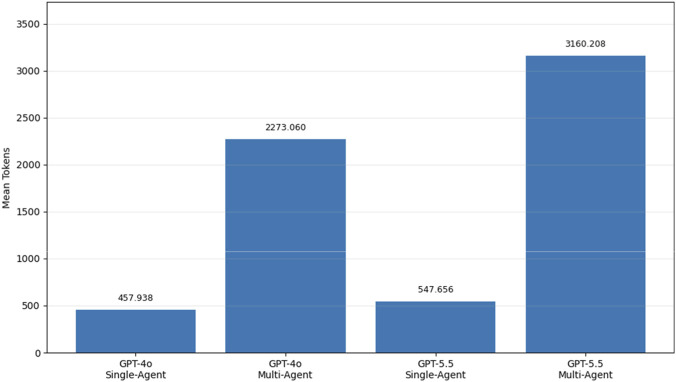
Mean token usage by model and architecture. Mean token usage is shown for the single-agent and multi-agent orchestration architectures across GPT-4o and GPT-5.5. Multi-agent orchestration consumed substantially more tokens than the single-agent baseline in both model conditions, indicating higher computational and cost overhead associated with multi-step coordinated reasoning.

Aggregate results are summarized visually in Figures 2–4, with full numeric values provided in Supplementary Table S1. Figure 2 reports decision accuracy, exact hazard F1, and semantic hazard F1. Figures 3, 4 report latency and token usage, respectively.

In the GPT-4o evaluation, the single-agent architecture achieved 0.810 decision accuracy, 0.081 exact hazard F1, and 0.131 semantic hazard F1. By comparison, GPT-4o multi-agent orchestration achieved 0.734 decision accuracy, 0.043 exact hazard F1, and 0.106 semantic hazard F1. The single-agent condition also required lower latency and token usage, averaging 2.32 s and 458 tokens per evaluation, compared with 11.83 s and 2,273 tokens for multi-agent orchestration.

In the GPT-5.5 evaluation, the single-agent architecture achieved 0.974 decision accuracy, 0.018 exact hazard F1, and 0.075 semantic hazard F1. By comparison, GPT-5.5 multi-agent orchestration achieved 0.934 decision accuracy, 0.000 exact hazard F1, and 0.060 semantic hazard F1. The single-agent condition again required lower latency and token usage, averaging 6.06 s and 548 tokens per evaluation, compared with 35.59 s and 3,160 tokens for multi-agent orchestration.

Across both model configurations, the single-agent architecture showed numerically higher decision accuracy and lower computational overhead than multi-agent orchestration. Hazard-label alignment remained limited across conditions, especially under exact matching, indicating that action selection and hazard-label fidelity should be interpreted as related but distinct evaluation outcomes. These results suggest that, in the tested OpenAI configurations and prompt-defined architecture, multi-agent orchestration increased latency and token usage without producing a corresponding improvement in aggregate decision accuracy or hazard F1.

### 4.3 Scenario-level and hazard-classification analysis

Scenario-level analysis focused on whether architecture choice changed the model’s ability to select the expected action and identify the expected hazard set across repeated evaluations. The corrected scoring approach distinguishes between exact canonical hazard matches, substring-based semantic matches, plausible non-canonical hazards, and spurious hazards. This distinction is important because an additional detected hazard may be operationally plausible even if it is not part of the scenario’s canonical expected_hazards list.

The aggregate hazard-classification results showed that multi-agent orchestration identified more hazards outside the canonical label set than the single-agent baseline. For GPT-4o, multi-agent orchestration produced a mean of 2.202 plausible non-canonical hazards and 3.312 spurious hazards per evaluation, compared with 1.376 plausible non-canonical hazards and 0.150 spurious hazards for the single-agent condition. For GPT-5.5, multi-agent orchestration produced a mean of 3.578 plausible non-canonical hazards and 2.506 spurious hazards per evaluation, compared with 2.392 plausible non-canonical hazards and 1.420 spurious hazards for the single-agent condition.

These findings indicate that multi-agent orchestration generated broader hazard lists than the single-agent architecture. Some of these additional hazards were plausible contextual observations, while others were classified as spurious because they lacked keyword overlap with the scenario context. This pattern helps explain why multi-agent orchestration sometimes showed broader hazard coverage while also producing lower precision and lower F1. The architecture did not simply miss all hazards; rather, it often expanded the hazard set beyond the benchmark’s expected labels.

Canonical hazard coverage showed a more nuanced pattern. In GPT-4o, multi-agent orchestration achieved higher canonical hazard coverage than the single-agent architecture, with means of 0.214 and 0.144, respectively. In GPT-5.5, canonical hazard coverage was also slightly higher for multi-agent orchestration, with means of 0.127 and 0.119, respectively. However, this broader coverage came with substantially higher false-positive counts and greater computational cost. As a result, canonical coverage alone does not indicate stronger decision-support performance unless considered alongside false positives, spurious hazards, latency, and token usage.

Overall, the scenario-level pattern suggests that the multi-agent architecture tended to surface more contextual risks but did not filter those risks as effectively into a concise final hazard set. This behavior is consistent with the design of the orchestration workflow: specialist agents evaluated the scenario from different perspectives, and the orchestrator synthesized their outputs. Without stronger hazard-filtering rules, this structure can preserve too many contextual concerns in the final output, reducing precision even when some additional observations are plausible.

### 4.4 Error analysis

**FIGURE 5:**
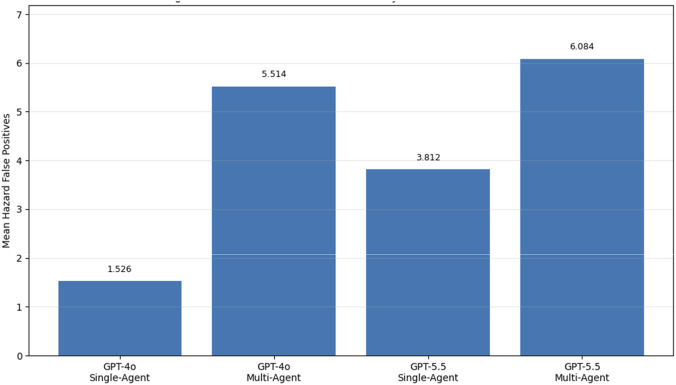
Mean hazard false positives by model and architecture. Mean hazard false-positive counts are shown for the single-agent and multi-agent orchestration architectures across GPT-4o and GPT-5.5. Higher values indicate a greater tendency to identify hazards beyond the canonical expected hazard set. Multi-agent orchestration produced more false-positive hazards than the single-agent architecture in both model conditions.

The error analysis indicates that the primary weakness of multi-agent orchestration was broader hazard over-identification rather than a uniform failure to select plausible final actions. Across both model configurations, the multi-agent condition produced more hazard false positives than the single-agent condition. Figure 5 summarizes this false-positive pattern across model and architecture conditions. For GPT-4o, multi-agent orchestration produced a mean of 5.514 false-positive hazards per evaluation, compared with 1.526 for the single-agent architecture. For GPT-5.5, multi-agent orchestration produced a mean of 6.084 false-positive hazards per evaluation, compared with 3.812 for the single-agent architecture.

This false-positive pattern contributed to lower hazard F1 scores under both exact and semantic scoring. In GPT-4o, exact hazard F1 decreased from 0.081 in the single-agent condition to 0.043 in the multi-agent condition, and semantic hazard F1 decreased from 0.131 to 0.106. In GPT-5.5, exact hazard F1 decreased from 0.018 in the single-agent condition to 0.000 in the multi-agent condition, and semantic hazard F1 decreased from 0.075 to 0.060.

The corrected results also show that the hazard-identification task remained difficult even after removing scoring-only fields from all model-facing prompts. Decision accuracy was substantially higher than hazard F1 across both model backends, indicating that the models were more reliable at selecting the expected operational action than at reproducing or semantically matching the expected hazard set. This distinction is important because a model may select the correct action while still over-identifying hazards, under-identifying canonical hazards, or using wording that only partially overlaps with the benchmark label set.

The results therefore support three observations. First, multi-agent orchestration increased hazard false positives relative to the single-agent baseline across both evaluated model configurations. Second, exact hazard F1 should be interpreted as strict label fidelity rather than complete operational hazard awareness. Third, semantic hazard F1 provides a more flexible substring-based measure of conceptual overlap, but it still does not fully substitute for expert human evaluation of hazard relevance. These distinctions address the need to separate canonical label matching, plausible non-canonical hazard detection, and truly spurious hazard generation.

### 4.5 Statistical comparisons

Scenario-level paired statistical comparisons were used to evaluate whether differences between the single-agent and multi-agent orchestration architectures were consistent across matched scenarios. To account for the repeated-run structure of the benchmark, the five repeated evaluations for each scenario were aggregated into scenario-level means before statistical testing. Each model comparison therefore used 100 paired scenario-level units rather than 500 run-level observations. Exact paired sign tests were conducted on these scenario-level pairs, Cohen’s dz was reported as the standardized paired-sample effect size, 95% bootstrap confidence intervals were generated by resampling paired scenarios, and Holm-Bonferroni correction was applied across 10 comparisons.

For GPT-4o, decision correctness numerically favored the single-agent architecture, with a scenario-level mean of 0.810 compared with 0.734 for multi-agent orchestration. The paired mean difference was 0.076, 95% CI [0.024, 0.130], with Cohen’s dz = 0.278, 95% CI [0.107, 0.432]. The unadjusted paired sign test was significant, p = 0.029, but the comparison did not remain significant after Holm-Bonferroni correction, adjusted p = 0.087. GPT-4o exact hazard F1 also numerically favored the single-agent architecture, 0.081 versus 0.043, with a paired mean difference of 0.038, 95% CI [0.011, 0.068], and Cohen’s dz = 0.261, 95% CI [0.090, 0.413], but the corrected comparison was not statistically significant, adjusted p = 0.401. GPT-4o semantic hazard F1 showed the same numerical direction, 0.131 versus 0.106, but was not significant, adjusted p = 0.560.

Efficiency effects for GPT-4o were stronger and remained statistically significant after correction. Mean latency was 2.32 s for single-agent and 11.83 s for multi-agent orchestration, yielding a paired mean difference of −9.51 s, 95% CI [−9.95, −9.13], with Cohen’s dz = −4.529, 95% CI [−6.624, −3.370]. Mean token usage was 458 tokens for single-agent and 2,273 tokens for multi-agent orchestration, yielding a paired mean difference of −1,815 tokens, 95% CI [−1,843, −1,787], with Cohen’s dz = −12.710, 95% CI [−14.350, −11.526]. Both efficiency comparisons had p < 0.001 and Holm-adjusted p < 0.001.

For GPT-5.5, decision correctness again numerically favored the single-agent architecture, with a scenario-level mean of 0.974 compared with 0.934 for multi-agent orchestration. The paired mean difference was 0.040, 95% CI [−0.004, 0.086], with Cohen’s dz = 0.173, 95% CI [−0.019, 0.328], and was not statistically significant, adjusted p = 0.238. GPT-5.5 exact hazard F1 favored the single-agent architecture, 0.018 versus 0.000, with a paired mean difference of 0.018, 95% CI [0.008, 0.030], and Cohen’s dz = 0.329, 95% CI [0.232, 0.433]. This comparison remained statistically significant after correction, p < 0.001, adjusted p = 0.001. GPT-5.5 semantic hazard F1 was numerically higher for the single-agent architecture, 0.075 versus 0.060, but did not remain statistically significant after correction, adjusted p = 0.130.

Efficiency effects for GPT-5.5 also strongly favored the single-agent architecture. Mean latency was 6.06 s for single-agent and 35.59 s for multi-agent orchestration, yielding a paired mean difference of −29.52 s, 95% CI [-30.22, −28.83], with Cohen’s dz = −8.253, 95% CI [−9.612, −7.337]. Mean token usage was 548 tokens for single-agent and 3,160 tokens for multi-agent orchestration, yielding a paired mean difference of −2,613 tokens, 95% CI [−2,658, −2,568], with Cohen’s dz = −11.393, 95% CI [−13.130, −10.255]. Both efficiency comparisons had p < 0.001 and Holm-adjusted p < 0.001.

Because the multi-agent condition necessarily required multiple specialist calls plus a final orchestration step for every scenario, the latency and token advantages of the single-agent architecture should be interpreted as an expected structural consequence of the tested design, with statistical testing quantifying the magnitude of that overhead rather than establishing it as an unexpected empirical discovery.

Overall, the scenario-level statistical comparisons support a more cautious interpretation than the original run-level analysis. The single-agent architecture showed consistent numerical advantages in decision correctness and hazard F1, but not all performance differences remained statistically significant after scenario-level aggregation and Holm-Bonferroni correction. The strongest and most consistent effects were observed for latency and token usage. These findings support the narrower conclusion that, in the tested OpenAI configurations and prompt-defined benchmark design, multi-agent orchestration introduced substantial computational overhead without producing a reliable improvement in aggregate decision accuracy or hazard F1 relative to the single-agent baseline.

### 4.6 Ablation analysis

To further examine the role of specialist agents within the multi-agent orchestration architecture, a GPT-4o ablation analysis was conducted by removing one specialist agent at a time from the orchestration workflow. The ablation conditions removed the telemetry agent, terrain agent, risk agent, or mission-planner agent while retaining the remaining specialists and the final orchestrator. Each ablation condition was evaluated across 500 run-level observations and summarized relative to the full GPT-4o multi-agent orchestration baseline using paired run-level McNemar comparisons matched by scenario_id and run_id. Because the ablation analysis was exploratory and limited to GPT-4o, these results are interpreted as role-contribution evidence rather than as a primary confirmatory statistical analysis.

The full GPT-4o multi-agent baseline achieved 0.734 decision accuracy, 0.043 exact hazard F1, 0.106 semantic hazard F1, 0.214 canonical hazard coverage, 11.83 s mean latency, and 2,273 mean tokens per evaluation. Removing the terrain agent produced the largest negative effect. The drop-terrain condition reduced decision accuracy to 0.666, a 6.8 percentage-point decrease relative to the full multi-agent baseline and reduced exact hazard F1 to 0.007. Canonical hazard coverage also declined from 0.214 to 0.119. The exact paired McNemar test comparing the drop-terrain condition with the full baseline indicated a statistically significant degradation in decision correctness, *p* < 0.001.

Removing the mission-planner agent produced the second-largest negative decision effect. The drop-mission-planner condition reduced decision accuracy to 0.684, a 5.0 percentage-point decrease relative to the full multi-agent baseline. Exact hazard F1 remained similar to the full baseline at 0.042, but canonical hazard coverage declined to 0.186. The exact paired McNemar test indicated that this reduction in decision correctness was statistically significant, *p* = 0.003, suggesting that the mission-planner agent contributed meaningfully to decision selection in the tested GPT-4o orchestration design.

The telemetry-agent and risk-agent ablations did not show the same negative decision effect. Removing the telemetry agent produced 0.744 decision accuracy, slightly higher than the full baseline, and this difference was not statistically significant, *p* = 0.551. Removing the risk agent produced 0.758 decision accuracy, also numerically higher than the full baseline, but the difference was not statistically significant, *p* = 0.104. These findings should not be interpreted as evidence that telemetry or risk reasoning is generally unnecessary. Rather, they suggest that, in this specific prompt-defined GPT-4o orchestration workflow, the standalone contribution of those agents was smaller or less consistently beneficial than the terrain and mission-planner roles.

The ablation ranking identified the terrain agent as the most important specialist role, followed by the mission-planner agent. The telemetry and risk agents had smaller or non-significant standalone effects in this configuration. These results help clarify that the multi-agent architecture did not fail uniformly across all specialist components. Instead, performance was more dependent on specific roles, particularly terrain interpretation and mission-planning synthesis. At the same time, all ablation conditions reduced latency and token usage relative to the full multi-agent baseline, reinforcing the broader finding that each additional specialist role carries a measurable computational cost. Because ablations were conducted only for GPT-4o, these results should be interpreted as exploratory evidence about specialist-role contributions within one model configuration rather than as a complete ablation study across all evaluated model backends.

These ablation results should also be interpreted relative to the single-agent baseline. Although the terrain and mission-planner ablations indicate that these specialist roles contributed to the full GPT-4o multi-agent workflow, the fully equipped multi-agent baseline still achieved lower decision accuracy than the GPT-4o single-agent condition (0.734 vs. 0.810). This pattern suggests that, in this prompt-defined workflow, specialist-role contributions did not fully offset orchestration overhead and may be consistent with coordination-related information dilution when scenario context is partitioned across intermediate agents and then recombined by an orchestrator.

## 5 Discussion

### 5.1 Architectural tradeoffs

The results indicate that increased agentic complexity did not improve overall decision-support performance in this benchmark. Across both evaluated OpenAI model configurations, the single-agent architecture showed numerically higher decision accuracy and substantially lower latency and token usage than multi-agent orchestration, although decision-accuracy differences did not remain statistically significant after scenario-level aggregation and Holm-Bonferroni correction. Hazard-identification performance remained limited across both architectures under exact and substring-based semantic scoring, but the single-agent architecture generally achieved higher hazard F1 scores and fewer false-positive hazards. This finding is important because multi-agent systems are often motivated by the assumption that decomposition and specialization will improve reasoning quality. In this study, however, decomposing rover decision support across telemetry, terrain, risk, and mission-planning agents did not produce a measurable advantage over a single model receiving the complete operational scenario context.

**FIGURE 6:**
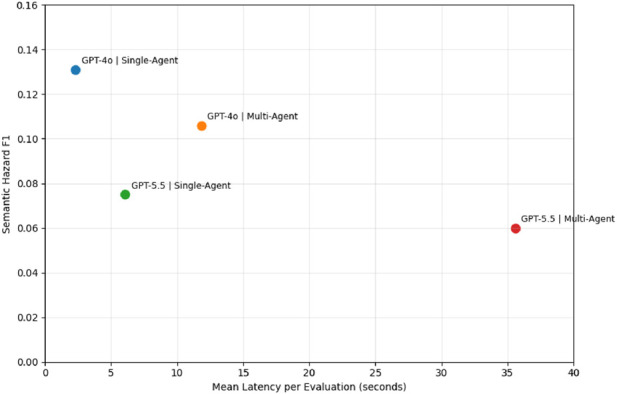
Latency versus semantic hazard F1. Relationship between mean latency and semantic hazard F1 across model and architecture conditions. The single-agent configurations occupy the more favorable region of the plot, with lower latency and higher semantic hazard F1 than the multi-agent orchestration configurations. This tradeoff visualization highlights that increased orchestration cost did not translate into improved hazard-identification performance in the present benchmark.

Figure 6 highlights the central tradeoff observed in the benchmark: the multi-agent orchestration architecture incurred substantially greater latency without a corresponding improvement in semantic hazard-identification performance.

One interpretation is that the task structure favored contextual coherence over decomposition. Each synthetic rover scenario contained a compact set of mission-relevant features, including telemetry, terrain description, environmental context, communication delay, mission objective, and mission log. Because these features were already available in a single structured context window, the single-agent architecture could reason across the full scenario without requiring inter-agent coordination. By contrast, the multi-agent architecture introduced intermediate analyses that the orchestrator had to reconcile. Rather than improving the final decision, these intermediate steps often increased the amount of risk-related language and produced additional hazards beyond the canonical expected set.

The GPT-5.5 results refine this interpretation. GPT-5.5 improved action-selection performance relative to GPT-4o under both architectures, but it did not eliminate the architectural tradeoff. The GPT-5.5 single-agent condition achieved 0.974 decision accuracy, compared with 0.934 for GPT-5.5 multi-agent orchestration. However, hazard F1 remained low in both GPT-5.5 conditions, with exact hazard F1 of 0.018 for single-agent and 0.000 for multi-agent orchestration. Semantic hazard F1 was also limited, with 0.075 for single-agent and 0.060 for multi-agent orchestration. These results indicate that stronger action selection does not necessarily imply precise hazard-label alignment or more efficient decision support.

The results therefore suggest that multi-agent orchestration may be most useful when the problem requires genuinely separable expertise, independent data access, tool execution, or long-horizon workflows that exceed the practical capabilities of a single model call. For compact decision-support scenarios with all relevant context available at once, a single-agent architecture may preserve coherence, reduce coordination overhead, and avoid unnecessary synthesis complexity.

### 5.2 Multi-agent coordination overhead

The multi-agent orchestration architecture incurred substantially greater latency and token usage across both model configurations. This was expected because the multi-agent condition required multiple specialist calls followed by final orchestration, but the magnitude of the overhead was substantial. In the GPT-4o run, multi-agent orchestration required 11.83 s per evaluation on average, compared with 2.32 s for the single-agent architecture. In the GPT-5.5 run, multi-agent orchestration required 35.59 s per evaluation, compared with 6.06 s for the single-agent condition.

Token consumption followed the same pattern. GPT-4o multi-agent orchestration used 2,273 mean tokens per evaluation, compared with 458 for the single-agent architecture. GPT-5.5 multi-agent orchestration used 3,160 mean tokens per evaluation, compared with 548 for the single-agent architecture. These results indicate that orchestration substantially increased computational cost without improving aggregate benchmark performance.

For Earth-based mission-support workflows, latency and token usage should be interpreted as operational overhead rather than automatic mission failure. This benchmark does not evaluate onboard flight control or real-time rover autonomy, and it does not define a mission-specific latency threshold. A response time of several tens of seconds may be acceptable in some asynchronous mission-log review workflows, but latency still affects throughput, analyst wait time, batch-processing capacity, cost, and scalability.

The coordination overhead also appears to have affected output precision. Specialist agents often surfaced plausible but non-canonical hazards, and the orchestrator frequently preserved or amplified those hazards rather than filtering them. This suggests that multi-agent orchestration can increase broader risk enumeration at the expense of precision when agents are not tightly constrained.

Overall, the results suggest that orchestration should be treated as a cost-bearing design choice rather than a default improvement. Multi-agent workflows may still be useful when agents perform genuinely distinct functions, use different tools, or access different data sources. However, when multiple agents reason over the same compact scenario context, the added coordination layer may increase cost and complexity without improving the final recommendation.

### 5.3 Hazard over-identification and failure modes

A notable pattern in the results was hazard over-identification. The multi-agent orchestration architecture frequently identified additional hazards beyond the expected canonical hazard set. Some of these additional hazards were plausible non-canonical observations, such as terrain instability, slope concerns, communication delay, wheel loading, or traction uncertainty. However, because they were not part of the expected hazard labels for the scenario, they reduced precision and lowered exact and semantic hazard F1.

This pattern was visible in the false-positive results. In the GPT-4o run, the multi-agent architecture produced a mean false-positive count of 5.514, compared with 1.526 for the single-agent architecture. In the GPT-5.5 run, the multi-agent architecture produced a mean false-positive count of 6.084, compared with 3.812 for the single-agent architecture. These results indicate that multi-agent orchestration amplified hazard inflation across both evaluated model configurations.

The decision-error pattern also requires careful interpretation. The earlier hypothesis that multi-agent orchestration errors would lean primarily toward conservative pause recommendations was not supported. In the corrected benchmark, the multi-agent architecture did not simply become more conservative in every decision case. Rather, its errors reflected a broader mismatch between hazard enumeration and final action selection. This is important because a system can list more hazards while still selecting an action that is not more conservative. Therefore, hazard inflation should not be interpreted as equivalent to safer behavior.

The GPT-5.5 results also show that stronger action-level performance can mask hazard-level weakness. Although GPT-5.5 achieved high decision accuracy in both architectures, the multi-agent architecture still produced lower hazard F1 and more false-positive hazards than the single-agent architecture. This distinction is important because mission-support systems must provide not only a final recommendation but also a reliable explanation of the hazards that justify that recommendation.

Overall, the results suggest that multi-agent decomposition can accumulate and preserve low-priority or secondary risk observations across specialist outputs. However, this pattern may reflect the specific orchestrator prompt design rather than an inherent limitation of all multi-agent architectures. In the tested workflow, the orchestrator primarily synthesized specialist outputs into a final recommendation. This aggregator-style design can behave similarly to a union operator by preserving many contextual risks raised by the specialist agents, including risks that are plausible but secondary or weakly supported.

A different orchestrator design that acts more explicitly as a critic or filter, using confidence thresholds, evidence requirements, or intersection-style agreement rules, may reduce hazard inflation. Future multi-agent rover decision-support systems should therefore distinguish primary hazards from secondary operational context, apply stricter grounding to detected hazards, and evaluate whether the orchestrator is designed to aggregate, arbitrate, or suppress unsupported hazard claims.

### 5.4 Implications for mission support systems

The findings do not imply that multi-agent systems are generally inferior to single-agent systems. Instead, they suggest that multi-agent orchestration should be justified by task requirements rather than adopted as a default architecture. For simulated Mars rover decision support, the tested orchestration design introduced latency, token cost, and hazard inflation without improving final decision quality. This result supports a more selective view of agentic architecture: the simplest architecture capable of producing reliable outputs may be preferable when the task is compact, structured, and fully represented in the input context.

For Earth-based mission-support workflows, single-agent systems may be useful for rapid triage of transmitted rover telemetry, mission logs, and terrain summaries. Multi-agent systems may still be valuable when different agents have access to distinct tools, datasets, or analytical procedures, such as image analysis, route optimization, power modeling, or scientific prioritization. In such cases, orchestration may provide value by coordinating genuinely different capabilities rather than asking multiple agents to reason over the same information.

The results also suggest that future multi-agent decision-support systems should include stronger arbitration and filtering mechanisms. Specialist outputs may need to distinguish between primary hazards, secondary contextual concerns, plausible non-canonical observations, and unsupported spurious hazards. Orchestrators may also require explicit rules for rejecting unsupported hazards, resolving agent disagreement, and preventing unnecessary escalation unless the evidence meets predefined thresholds.

Finally, this benchmark demonstrates the importance of evaluating agent architectures empirically rather than assuming that more agents produce better reasoning. In constrained autonomous or mission-support domains, architectural performance must be assessed jointly across accuracy, hazard identification, latency, token usage, and failure behavior. The central contribution of this study is therefore not that single-agent systems will always outperform multi-agent systems, but that architectural complexity carries measurable costs that must be justified by measurable improvements.

### 5.5 Limitations

This study was designed as a controlled synthetic benchmark before extending the framework to image-grounded Mars rover decision-support scenarios. The synthetic design allowed the same action categories, hazard labels, architecture conditions, and repeated-run structure to be tested under reproducible conditions. However, the scenario labels reflect a predefined synthetic decision policy rather than expert-validated rover operations data. Although the scenario-generation process was designed to reduce *post hoc* author adjustment, the labels were not independently reviewed by rover operators, planetary scientists, or multiple human annotators. Future validation with independent annotators or domain experts would strengthen the benchmark and improve interpretation of ambiguous rover decision cases.

The benchmark also used deterministic exact and substring-based semantic hazard scoring. Exact hazard F1 measures fidelity to canonical hazard labels, while semantic hazard F1 measures substring-based conceptual overlap. These metrics improve reproducibility, but they do not fully capture expert judgment about whether an additional hazard is operationally useful, redundant, or genuinely spurious. Human expert review would be needed to validate hazard relevance more rigorously.

This study did not define or validate a mission-specific standard operating procedure latency threshold. The latency analysis therefore evaluates relative computational and workflow overhead rather than declaring a universal operational cutoff for Earth-based rover decision support. A response time of several tens of seconds may be acceptable in some asynchronous review workflows, but less suitable for high-throughput triage, time-sensitive review, or large-scale batch processing.

The model comparison was limited to two OpenAI model backends and one prompt-defined multi-agent orchestration design. Therefore, the findings should be interpreted as applying to the tested OpenAI configurations and supervisor-style orchestration workflow, not to all LLM providers, model versions, or multi-agent systems. Heterogeneous multi-agent systems, tool-using agents, domain-adapted models, or specialist models trained for rover telemetry and terrain interpretation may produce different results.

Finally, the ablation analysis was conducted only for the GPT-4o condition due to API cost and runtime constraints. As a result, ablation findings should be interpreted as exploratory evidence about specialist-role contributions within the GPT-4o multi-agent configuration, not as a complete ablation analysis across all model backends.

### 5.6 Future work

Future work should extend this benchmark to human-annotated Mars imagery and terrain-derived decision records, allowing architecture comparisons to be tested against more realistic perception inputs and independently reviewed labels. Expanding the scenario corpus beyond 100 synthetic records and adding independent human review of expected actions and hazards would strengthen label validity and clarify whether model disagreement reflects error or scenario ambiguity.

Additional work should compare deterministic hazard scoring against human annotation and more advanced semantic evaluation methods. Future metrics should distinguish canonical hazard coverage, plausible non-canonical observations, secondary monitoring concerns, and unsupported spurious hazards. Finally, future studies should evaluate broader model sets and multi-agent designs, including GPT-5.5 ablations, heterogeneous multi-agent systems, and alternative orchestration architectures.

## 6 Conclusion

This study evaluated single-agent and multi-agent orchestration architectures for simulated Mars rover decision support using 100 synthetic mission-inspired scenarios with five repeated runs per condition. The benchmark compared OpenAI GPT-4o and GPT-5.5 using repeated scenario evaluations and scenario-level paired statistical comparisons. The reported results are based on a no-label-leakage workflow in which expected actions and expected hazards were reserved for evaluator-side scoring only.

Across the tested configurations, the single-agent architecture showed numerical advantages in decision accuracy, hazard-label alignment, and hazard false-positive counts, while requiring substantially lower latency and token usage than multi-agent orchestration. However, decision-quality differences were not consistently significant after scenario-level analysis and multiple-comparison correction. Hazard-identification performance remained challenging across both architectures, especially under strict canonical-label and substring-based semantic scoring.

The findings do not show that multi-agent orchestration is generally ineffective. Rather, they show that, within this synthetic benchmark and prompt-defined supervisor-style orchestration design, additional agent decomposition did not provide enough reliable performance benefit to justify its added latency, token cost, and hazard over-identification. Multi-agent systems may still be useful when agents have access to distinct tools, data sources, sensing modalities, external APIs, or domain-specialized capabilities. However, in short-context, tool-less, static decision-support tasks where all relevant context is already available in a compact scenario, a single-agent architecture may provide a stronger balance of efficiency, coherence, and output precision.

This study contributes a reproducible architecture-level benchmark for examining when LLM-based orchestration is worth its operational cost in mission-inspired decision-support settings. It also highlights the importance of separating model-facing inputs from scoring labels, documenting prompts and model-facing fields, and distinguishing canonical hazard fidelity from broader operational hazard awareness. Future work should extend this benchmark to human-annotated Mars imagery, terrain-derived decision records, additional model providers, heterogeneous multi-agent systems, and expert-reviewed hazard evaluation.

## Data Availability

The datasets presented in this study can be found in online repositories. The names of the repository/repositories and accession number(s) can be found below: https://doi.org/10.5281/zenodo.20563739.

## References

[B1] Abcouwer N. Daftry S. Del Sesto T. Toupet O. Ono M. Venkatraman S. (2021). Machine learning based path planning for improved rover navigation. IEEE Aerosp. Conf. 50100, 1–9. 10.1109/aero50100.2021.9438337 34713276

[B2] Arzo S. T. Sikeridis D. Devetsikiotis M. Granelli F. Fierro R. Esmaeili M. (2023). Essential technologies and concepts for massive space exploration: challenges and opportunities. IEEE Trans. Aerosp. Electron. Syst. 59 (1), 3–29. 10.1109/taes.2022.3169126

[B3] Barnell M. Raymond C. Loomis L. Isereau D. Brown D. Vidal F. (2023). Advanced ultra low-power deep learning applications with neuromorphic computing, in 2023 IEEE High Performance Extreme Computing Conference (HPEC), 1–4. 10.1109/hpec58863.2023.10363561

[B4] Beegle L. Bhartia R. White M. DeFlores L. Abbey W. Wu Y.-H. (2015). SHERLOC: scanning habitable environments with raman and luminescence for organics and chemicals. IEEE Aerosp. Conf., 1–11. 10.1109/aero.2015.7119105

[B5] Bommasani R. Hudson D. A. Adeli E. Altman R. Arora S. von Arx S. (2021). On the opportunities and risks of foundation models. arXiv Preprint arXiv:2108.07258. Available online at: https://arxiv.org/abs/2108.07258 (Accessed June 26, 2026).

[B6] Brockers R. Delaune J. Proenca P. Schoppmann P. Domnik M. Kubiak G. (2021). Autonomous safe landing site detection for a future Mars science helicopter. IEEE Aerosp. Conf. 50100, 1–8. 10.1109/aero50100.2021.9438289 34713276

[B7] Brown T. B. Mann B. Ryder N. Subbiah M. Kaplan J. Dhariwal P. (2020). Language models are few-shot learners. Adv. Neural Inf. Process. Syst. 33, 1877–1901. Available online at: https://arxiv.org/abs/2005.14165 (Accessed June 26, 2026).

[B9] Das M. Mandal S. Roy P. Roy A. Mukherjee A. Mahala S. (2025). Design and implementation of multi-agent systems: a lang Graph-based approach, in 2025 International Conference on Engineering Innovations and Technologies (Icoeit). 10.1109/ICOEIT63558.2025.11211513

[B10] Dimakos A. Woodhall D. Asif S. (2021). A study on centralized and decentralized swarm robotics architecture for part delivery system. Acad. J. Eng. Stud. 2 (3). 10.31031/aes.2021.2.000540

[B11] Du H. Nguyen H. Thudumu S. Vasa R. Mouzakis K. (2026). Goal-oriented multi-agent reinforcement learning for decentralized agent teams, in 2026 IEEE 23rd Consumer Communications and Networking Conference (CCNC). 10.1109/CCNC65079.2026.11366321

[B12] Ferreira P. V. Paffenroth R. Wyglinski A. M. Hackett T. M. Bilen S. G. Reinhart R. C. (2017). Multi-objective reinforcement learning-based deep neural networks for cognitive space communications. 2017 Cognitive Commun. Aerosp. Appl. Workshop (CCAA), 1–8. 10.1109/ccaaw.2017.8001880

[B13] Geng S. Cooper H. Moskal M. Jenkins S. Berman J. Ranchin N. (2025). Generating structured outputs from language models: benchmark and studies. arXiv preprint arXiv:2501.10868. Available online at: https://arxiv.org/abs/2501.10868.

[B14] Huang Q. Vora J. Liang P. Leskovec J. (2023). MLAgentBench: evaluating language agents on machine learning experimentation. arXiv preprint arXiv:2310.03302. Available online at: https://arxiv.org/abs/2310.03302.

[B15] Liu X. Yu H. Zhang H. Xu Y. Lei X. Lai H. (2023). AgentBench: evaluating LLMs as agents. arXiv preprint arXiv:2308.03688. Available online at: https://arxiv.org/abs/2308.03688.

[B16] Liu A. Hill C. Jiang J. Zhuo Z. (2025). Can reasoning LLMs eliminate conformity in multi-agent systems?. in 2025 IEEE International Conference on Data Mining Workshops (ICDMW). 10.1109/ICDMW69685.2025.00406

[B17] Maki J. N. Gruel D. McKinney C. Ravine M. A. Morales M. Lee D. (2020). The Mars 2020 engineering cameras and microphone on the perseverance rover: a next-generation imaging system for Mars exploration. Space Sci. Rev. 216 (8), 137. 10.1007/s11214-020-00765-9 33268910 PMC7686239

[B18] Murbach M. Salas A. (2022). Techedsat-13: the first flight of a neuromorphic processor. NASA Tech. Rep. Serv. (NTRS). Available online at: https://ntrs.nasa.gov/citations/20220005780.

[B19] Omar A. Farag M. Alhamad R. (2024). Swarm robotics: a new paradigm in robotic space exploration. IAF Space Explor. Symp., 29–31. 10.52202/078357-0005

[B20] Qin Y. Liang S. Ye Y. Zhu K. Yan L. Lu Y. (2023). ToolLLM: facilitating large language models to master 16000+ real-world APIs. arXiv preprint arXiv:2307.16789. Available online at: https://arxiv.org/abs/2307.16789.

[B21] Sasaki T. Otsu K. Thakker R. Haesaert S. Agha-mohammadi A. (2020). Where to map? Iterative Rover-copter path planning for Mars exploration. IEEE Robotics Automation Lett. 5 (2), 2123–2130. 10.1109/lra.2020.2970650

[B22] Vaswani A. Shazeer N. Parmar N. Uszkoreit J. Jones L. Gomez A. N. (2017). Attention is all you need. Adv. Neural Inf. Process. Syst. 30. Available online at: https://arxiv.org/abs/1706.03762.

[B23] Verma V. Maimone M. W. Gaines D. M. Francis R. Estlin T. A. Kuhn S. R. (2023). Autonomous robotics is driving perseverance rover’s progress on Mars. Sci. Robotics 8 (80), eadi3099. 10.1126/scirobotics.adi3099 37494463

[B24] Wilhelm T. Kobmann D. Wohler C. (2022). Machine learning on Mars: open challenges, similarities and differences to Earth remote sensing. IGARSS 2022 - 2022 IEEE Int. Geoscience Remote Sens. Symposium, 5373–5376. 10.1109/igarss46834.2022.9884096

[B25] Wu Q. Bansal G. Zhang J. Wu Y. Li B. Zhu E. (2023). AutoGen: enabling next-gen LLM applications *via* multi-agent conversation. arXiv preprint arXiv:2308.08155. Available online at: https://arxiv.org/abs/2308.08155.

[B26] Yao S. Zhao J. Yu D. Du N. Shafran I. Narasimhan K. (2022). ReAct: synergizing reasoning and acting in language models. arXiv preprint arXiv:2210.03629. Available online at: https://arxiv.org/abs/2210.03629.

