# Supplementary material for: OpenAI single-agent LLM architecture reduces computational overhead relative to multi-agent orchestration in a simulated mars rover decision-support benchmark
Source: Front Robot AI. 2026 Jul 6;13:1877762. doi: 10.3389/frobt.2026.1877762 (PMC13381255; doi:10.3389/frobt.2026.1877762)
Supplement: Supplementary file 1 [file Supplementaryfile1.pdf]

Supplementary material

Supplementary Table S1. Cross-model aggregate architecture comparison

Cross-model aggregate architecture comparison for the single-agent and multi-agent orchestration architectures across GPT-4o and GPT-5.5 model configurations. Each model-architecture condition was evaluated across 100 synthetic Mars rover scenarios with five repeated evaluations per scenario. Metrics include decision accuracy, exact hazard F1, semantic hazard F1, mean hazard false-positive count, mean latency per evaluation, and mean token usage per evaluation.

Model	Architecture	Runs	Scenarios	Evaluations	Decision Accuracy	Exact Hazard F1	Semantic Hazard F1	Mean False Positives	Mean Latency (s)	Mean Token Usage
**GPT-4o**	Single-Agent	5	100	500	0.810	0.081	0.131	1.526	2.32	458
**GPT-4o**	Multi-Agent	5	100	500	0.734	0.043	0.106	5.514	11.83	2,273
**GPT-5.5**	Single-Agent	5	100	500	0.974	0.018	0.075	3.812	6.06	548
**GPT-5.5**	Multi-Agent	5	100	500	0.934	0.000	0.060	6.084	35.59	3,160

S1. Label-Leakage Verification

To verify that the revised benchmark workflow removed scoring-label visibility from model-facing prompts, we conducted a run-level check for verbatim canonical hazard reproduction. In this check, a verbatim match was operationalized as exact_hazard_f1 = 1.0, indicating that the model response reproduced the full canonical expected hazard set for that evaluation.

The revised no-leakage run-level outputs showed near-zero verbatim canonical hazard reproduction across architectures and model backends. In the GPT-4o single-agent condition, verbatim matches occurred in 6 of 500 evaluations, corresponding to a rate of 1.2%. In the GPT-5.5 single-agent condition, verbatim matches occurred in 0 of 500 evaluations, corresponding to a rate of 0.0%. The multi-agent orchestration condition produced 0 of 500 verbatim matches for both GPT-4o and GPT-5.5, corresponding to 0.0% in each case.

These values contrast with the original submission, where the single-agent condition reproduced the canonical expected hazard set in 84.6% of GPT-4o runs and 35.0% of GPT-5.5 runs. The revised results therefore indicate that canonical hazard reproduction dropped to near-zero levels after the expected_action and expected_hazards fields were removed from all model-facing prompts and retained only for evaluator-side scoring. The run-level files deposited with the revised Zenodo record correspond to these no-leakage runs and are the files used to generate the revised manuscript tables.

Supplementary Table S2. Run-level verbatim canonical hazard reproduction rates under the finalized no-label-leakage workflow.

Model	Architecture	Verbatim Matches	Evaluations	Verbatim-Match Rate
**GPT-4o**	Single-agent	6	500	1.2%
**GPT-4o**	Multi-agent orchestration	0	500	0.0%
**GPT-5.5**	Single-agent	0	500	0.0%
**GPT-5.5**	Multi-agent orchestration	0	500	0.0%

Supplementary File 1

Prompt templates, model-facing fields, output schema, run metadata, no-leakage run-level outputs, and scenario-level statistical analysis files are available in the archived Zenodo repository, version 1.1.0: https://doi.org/10.5281/zenodo.20563739.
